# Home health care professionals’ experiences of working in integrated teams during the COVID-19 pandemic: a qualitative thematic study

**DOI:** 10.1186/s12875-022-01934-1

**Published:** 2022-12-14

**Authors:** Lina Emmesjö, Jenny Hallgren, Catharina Gillsjö

**Affiliations:** 1 School of Health Sciences, University of Skövde, P.O. Box 408, SE-541 28 Skövde, Sweden; 2 School of Health and Welfare, Jönköping University, Jönköping, Sweden; 3 College of Nursing, University of Rhode Island, Kingston, RI USA

**Keywords:** COVID-19, Home health care, Home health care physician, Registered nurse, Municipal care, Thematic analysis, Qualitative

## Abstract

**Background:**

Since COVID-19 emerged, over 514 million COVID-19 cases and 6 million COVID-19-related deaths have been reported worldwide. Older persons receiving home health care often have co-morbidities that require advanced medical care, and are at risk of becoming severely ill or dying from COVID-19. In Sweden, over 10,000 COVID-19-related deaths have been reported among persons receiving municipal home health and social care. Home health care professionals have been working with the patients most at risk if infected. Most research has focused on the experiences of professionals in hospitals and assistant nurses in a home care setting. It is therefore valuable to study the experiences of the registered nurses and physicians working in home health care during the COVID-19 pandemic to learn lessons to inform future work.

**Method:**

A thematic qualitative study design using a semi-structured interview guide.

**Results:**

The health care professionals experienced being forced into changed ways of working, which disrupted building and maintaining relationships with other health care professionals, and interrupted home health care. The health care professionals described being forced into digital and phone communication instead of in-person meetings, which negatively influenced the quality of care. The COVID-19 pandemic brought worry about illness for the health care professionals, including worrying about infecting patients, co-workers, and themselves, as well as worry about upholding the provision of health care because of increasing sick leave. The health care professionals felt powerless in the face of their patients’ declining health. They also faced worry and guilt from the patients’ next of kin.

**Conclusion:**

Home health care professionals have faced the COVID-19 pandemic while working across organizational borders, caring for older patients who have been isolated during the pandemic and trying to prevent declining health and feelings of isolation. Due to the forced use of digital and phone communication instead of in-person visits, the home health care professionals experienced a reduction in the patients’ quality of care and difficulty maintaining good communication between the professions.

## Background

Coronavirus disease (COVID-19), was declared a pandemic on March 11, 2020 by the World Health Organization [1]. As of May 11, 2022, over 514 million confirmed cases of COVID-19 have been reported and more than 6 million COVID-19-related deaths [2]. Persons aged above 60 years and those with underlying medical problems, such as high blood pressure, heart and lung issues, and diabetes, are at higher risk of COVID-19 infection developing into a serious illness with an increased risk of hospitalization and death [3]. Older persons living at home and receiving home health care are often frail, with several diagnoses, and require advanced medical care [4, 5], thereby being at risk of contracting the virus and becoming severely ill or dying if infected [6]. Older persons have also been denied health care and scarce medical resources during the pandemic due to their age [7–10]. As of March 28, 2022, over 10,000 deaths related to the COVID-19 pandemic have been reported among persons receiving municipal health and social care in Sweden [11].

In Sweden, most of the COVID-19 deceased have been over 85 years old [11]. Sweden has not had a strict lockdown, but have focused on social distancing, increased hygiene, and use of face shields and masks. Restaurants and shops have remained open with restrictions limiting the number of persons and the opening hours. The public has been recommended to restrict socializing to a certain number of persons. Citizens have been recommended to work from home but allowed to attend their workplaces if needed. Stricter restrictions during specific time periods have been applied to persons over 70 years old, who have been instructed to stay inside and avoid large gatherings and in-person contact as much as possible [12]. Between 44 and 60% of persons over 70 years old have reported refraining from seeking health care during the pandemic, even when required [13].

The municipalities in Sweden are responsible for health and social care for older persons with complex care needs that can be resolved by a registered nurse (RN). Since the municipality is not allowed to employ physicians, primary health care is responsible for medical care needing a physician [14, 15]. Sweden has more practicing registered nurses and physicians than the European average. However, even before the COVID-19 pandemic, municipalities struggled with employing registered nurses due to a nationwide shortage of registered and specialized nurses, which the municipalities self-report [13]. Before the pandemic, home health care personnel in municipalities often had uncertain employment status with much movement between employers. They also meet between 15 and 20 older persons per working day [12], having the possible risk of rapidly spreading the virus if infected themselves. In relation, an older person admitted to home health care may have had, on average, 16 different careers during a 2-week period [12], which could be seen as problematic when trying to lower the number of persons met during the pandemic to avoid possible contamination [16]. To meet the need for home health care, a county in Sweden created the Mobile Integrated Care Model (MICM).

The MICM with home health care physicians is an interdisciplinary care model with a municipality-specific health care team. The team comprises registered nurses (RNs), physiotherapists, occupational therapists, and assistant nurses (ANs) employed by the municipality. The MICM-physician is employed by the region through a primary health care center. The health care aims to enhance quality of life, continuity, and accessibility for the patients and their next of kin, as well as to be perceived as coherent, although provided by different health care authorities. The health care is intended to be grounded in person-centered care [17, 18], with a prerequisite partnership between the health care personnel and the patient [19, 20]. The MICM with a home health care physician has been implemented in a region with 49 municipalities in Sweden. It has been applied in different ways, but all include an MICM-physician and a municipality RN making home visits to patients at least once a year to co-create a medical health care plan. During the COVID-19 pandemic, MICM health care professionals evaluated patients admitted to home health care who showed symptoms of COVID-19 and cared for them if infected.

During the pandemic, health care professionals working in hospitals and primary health care experienced heavier workloads, lower general health, and increased pressure [21], higher rates of anxiety than before the pandemic, loneliness, anger, fear of death, and of transmitting the disease [22]. Similarly, RNs experienced higher workloads than other health care professionals [21] and moral distress related to the work environment, quality of care, and patient safety, to the point of considering leaving their employment [23, 24]. Studies have also shown that ANs working in home care experienced fear of infection, lack of guidance, unsafe hospital discharges, and staff shortages [25]. Health care professionals working in home health care experienced increased anxiety, depression, stress, and insomnia [26], having worked with the patient group most at risk if infected with COVID-19. Furthermore, home health care personnel were at risk of becoming infected because of inconsistent information and regulations throughout the pandemic, while perceiving themselves as invisible in media coverage [27]. Research on the experiences of health care professionals working in home health care is limited, especially in relation to integrated care models. Most research has focused on the experiences of hospital personnel and ANs in a home care setting. It is therefore relevant to study the experiences of RNs and physicians working in MICM during the COVID-19 pandemic to reveal lessons learned that can inform future work.

## Aim

To describe the experiences of RNs and MICM-physicians working in home health care in an integrated care model during the COVID-19 pandemic.

## Method

A qualitative exploratory design with thematic analysis based on Braun and Clark [28, 29] was chosen.

### Participants

The RNs were recruited via the heads of departments for health and social care of eight municipalities. The MICM-physicians were recruited via the executive director of two primary health care territories and unit managers in two private primary health care centers. The participants were eligible for inclusion if they had worked in home health care for at least 6 months. Eight municipalities of varying sizes were involved. The researchers were not informed if any RNs declined to participate, but two MICM-physicians declined because of external circumstances. The participating health care professionals received oral and written information about the study that described the aim, the method, and the researchers’ credentials. Eight RNs and six MICM-physicians agreed to participate (Table 1).

**Table 1:** Participant characteristics

Occupation	Age	Women	Men	Work experience
Registered Nurses *n* = 8	30–60	7	1	6–38 years
MICM-Physicians *n* = 6	37–68	3	4	12–45 years

### Data collection

An inductive approach was chosen for the interviews to capture the participants’ thoughts and experiences of working in home health care during the COVID-19 pandemic. Semi-structured interviews were conducted using an interview guide with open-ended questions. The interviews lasted between 20 and 72 minutes, were voice recorded, and transcribed. The interviews took place at a location chosen by the participants. Two preferred their own home, while twelve chose their workplace. Three interviews were conducted digitally and eleven face-to-face starting September 2020 through April 2021. The researcher conducting the interviews collected field notes in the form of reflections.

### Data analysis

The data were analyzed following the thematic analysis steps of Braun and Clark [28, 29] (Table 2), deemed relevant to the data because of the possibility to provide rich, detailed account of complex data [29]. The thematic analysis was iterative, and the authors identified, summarized, and interpreted the explicit and latent patterns of meanings addressing the research aim within and across the data. The analysis started with reading the transcripts and field notes several times to become familiar with the data and search for patterns of meanings. Next, data relevant to the aim were extracted, and 13 initial codes, both explicit and latent, were identified across the data set. The codes were read several times and sorted into 8 potential themes with the data extracts related to each code. The themes were then cross-checked with the entire data set, resulting in three main themes and six sub-themes (Table 3). Quotes were chosen to illustrate the findings.

**Table 2:** Thematic analysis process

Phases	Descriptions
Phase 1: Familiarization	The transcribed interviews and field notes were read several times, and the research team shared and commented on their initial reflections.
Phase 2: Coding	The first author extracted the data relevant to the aim and conducted the initial inductive coding. Both explicit and latent data were coded across the data set. 13 initial codes were identified and discussed: home visits, paused ways of working because of the pandemic, supporting the patient, worry among the personnel, removal of social places, patients’ mental health, protective gear/restrictions, testing, digital and phone solutions, shifting directives, vaccination, the future, and next of kin.
Phase 3: Searching for themes	Codes were sorted and reviewed, placed into a thematic map, and discussed by the authors. The detailed codes and hierarchies between them were discussed. The codes with their extracted data were sorted into 8 potential themes.
Phase 4: Reviewing themes	The authors reviewed and refined the 8 potential themes in relation to the codes and the extracted data. The themes were checked across the data set. During the cross-checking, overlapping themes were merged. The themes were refined and reworked into 3 main themes and 7 subthemes.
Phase 5: Defining and naming the themes	The themes were defined and refined in light of the codes and collated data extracts and triangulated in team meetings to reach consensus regarding the themes. The themes were defined and named, and the core content of each theme was described in text.
Phase 6: Producing the report	The final write-up of the analysis. All the authors participated in debriefing, interpretation, coding, and analysis of the data, including checking and critically revising the final version of the paper.

**Table 3:** Findings

Theme	Sub-theme
Forced into a changed way of working	*Avoiding ‘unnecessary’ visits led to disrupted relationships with patients*
	*Forced to pause usual work; development of health care on hold*
	*Forced to use digital and phone communication, which influenced the quality of care*
Worry about illness brought into the work setting	*Worry about infecting patients, co-workers, and oneself*
	*Worry about maintaining provision of care due to colleagues’ increased sick leave*
Trying to bridge the gap of patients’ isolation	*Powerless in the face of declined health for isolated patients*
	*Meeting increased worry and guilt from the next of kin*

### Ethical considerations

The project was approved by the Ethical Review Authority (Dnr 1020-17; 2019-02563; 2020-04324) and conducted according to the Declaration of Helsinki [30]. The participants received oral and written information about the project and were informed that participation was voluntary and could be ended at any time without consequence. Informed consent was obtained from all the participants.

## Findings

The thematic analysis revealed three main themes and seven sub-themes (Table 3).

### Forced into a changed way of working

The RN and MIM-physicians working in MICM were forced to change their way of working because of the pandemic. These changes included pausing usual work tasks, such as making home visits. Maintaining relationships with patients became difficult because of this pause. Furthermore, the work to structurally improve health care was put on pause, since the priority was the COVID-19 pandemic. The RN and MICM-physicians also described having to change the way they communicated from in-person to digital or phone communication. The forced changes were described as challenges that impacted the quality of care, since making health care decisions became harder.

#### Avoiding ‘unnecessary’ visits led to disrupted relationships with patients

RN and MICM-physicians working in MICM were asked to pause their usual work and prioritize working in a way suited to a pandemic. According to the participants, this entailed only making necessary home visits to the patients, which disrupted their relationships with them. One RN said:” *We’ve been told not to do unnecessary visits, but what is an unnecessary visit?”* Because of this, it was the individual RN or MICM-physician who had to evaluate which visits were necessary or not. The forced pause made creating and maintaining relationships with patients difficult, since the participants could not use their usual strategies. One MICM-physician said: *“I feel limited, and I’m used to being close to my patients and I like that. I want to create a relationship and that’s much more difficult, and I don’t feel comfortable with that.”* Frequent visits to patients, which ceased because of the forced pause, had created security and maintained relationships. Instead a form of cluster care was implemented, where fewer visits were conducted. Another way of creating security and building trust with patients was through touch, which also had to pause because of the pandemic. One RN said: “*You have to keep distance, and I like being close to my patients. I believe in closeness and giving them a hug. You can’t be close now*.” The MICM-physicians were supposed to meet all patients at least once a year on a rolling schedule, but because of the pandemic, several MCIM physicians decided to pause these visits. The RNs explained that the MICM-physician became invisible for the patients when only making necessary home visits. The individual MICM-physician decided what was “necessary,” which varied between only making acute visits to continuing to make medical health care plans and created friction between the RNs and MICM-physicians. The MICM-physicians who decided to pause creating medical health care plans remarked that it worked for a limited time but could not continue for longer. The MICM-physicians who continued to make home visits explained that not making them threatened patient safety and disrupted the relationship with patients. Some RNs said that the patients did not notice the lack of home visits from the MICM-physician. Other RNs claimed that conflicts had arisen between the RN and the MICM-physician since patients were angry about the lack of MICM-physician contact. The participants had different views on whether the patients were provided with the health care they needed due to the paused home visits and disrupted relationships. The participants commented that health care became need-oriented rather than based on relationships and person-centered.

#### Forced to pause usual work; development of health care on hold

New projects planned for 2020 could not be prioritized, and the RNs described having to prioritize any possible situation arising out of the pandemic instead of improving health care and working with things such as fall prevention, nutritional status or structure of medicine safety. One RNs said: *“Much work has been paused just so you can work with COVID and prepare, and just bringing the work forward has paused.”* Health care plans and visions, which sometimes had been prepared years in advance, were paused to focus on preparing for and working with COVID-19 which was described to possibly impact the safety of the everyday health care. The participants hoped that there would be a before and after COVID-19. They expressed the hope that their patients and the population in general would be vaccinated so that the organization and provision of health care in the MICMs could go back to working as before and continue developing health care. Other participants did not think that the pandemic would end, and even if it did, the world would be changed forever. They all hoped to be able to start again after the pandemic with the plans and visions that had been paused.

#### Forced to use digital and phone communication, which influenced the quality of care

The participants explained that they had to communicate differently with each other due to the COVID-19. Rounds, where RN and MICM-physicians met to discuss patient health care, previously conducted in person changed to phone or video calls, dictated by the individual MICM-physician. The RN and MICM-physicians stated that phone or video rounds did not work as well as in-person rounds and that it was hard to make patient evaluations together digitally. Misunderstandings also arose when the participants could not judge colleagues’ body language, which was described both during phone and video calls. Digital rounds were described as leading to more work than in-person ones because of the miscommunication they resulted in and how the RN could not see the MICM-physician’s screen, an obstacle to quality health care, according to the RNs. Some MICM-physicians working from home felt that digital communication worked well and did not see many downsides. The RNs and MICM-physicians said that not only rounds but also most communication was over the phone, due to avoiding unnecessary meetings with each other. Holding rounds on the phone was seen as more challenging than digital ones, according to the participants. One RNs said: *“The physician works from home. She’s there on the phone. You’re supposed to meet as few people as possible, and in health care you need to meet, to have physical meetings. It’s much harder to reach good quality during the pandemic.”*

The RNs said that they changed their ways of working by trying to keep in contact with patients over the phone, resulting in challenges that physical meetings did not have, influencing quality of care. One RN said: *“Many times you might call instead, but it’s hard because the patients have bad hearing, and they try to hear you, but no one ever hears me on the phone.”* Evaluating patients over the phone was described as a challenge that influenced the quality of care, since the participants could not make evaluations as before the pandemic.

Team meetings, including other health care professionals than the RN and the MICM-physician, and personnel meetings within the municipality had to be held digitally or were canceled. The RNs observed that the digital meetings were more efficient than those conducted in-person, and that the focus of the meeting became the only thing discussed. However, the lack of other topics was seen as problematic at times, as one RN said: *“The chit-chat can be a dialogue too, where new things about the patient come forward.”* Meeting in person was often preferred in meetings within the municipality, since discussions were more open. In some smaller municipalities, they continued having in-person meetings, since they shared staff rooms and office spaces. RNs in some municipalities met with the assistant nurses (AN) in person less frequently to avoid infection. Not seeing each other as often as before led to more phone calls and miscommunication, which impacted the quality of health care, explained the RNs. Other RNs changed the way they worked and made sure to visit the AN group several times a day to ensure that the ANs felt supported by the RNs. The participants noted that they had no choice but to work this way. Digital and phone communication was something they simply had to use, even if they thought that they did not work optimally and negatively impacted the quality of health care.

### Worry about illness brought into the work setting

The COVID-19 pandemic brought worry about illness into the work setting. The RN and MICM-physicians worried about infecting patients, co-workers, and themselves, as they initially worked without protective gear. The constantly changing regulations were followed as closely as possible, which eased worries about infection. The participants worked closely with the ANs to increase their knowledge and ease their worries about COVID-19. Worry about illness also led to worry about providing care, since sick leave increased among the RNs and ANs. This disrupted continuity for the patients, since the RNs and ANs had to reach more patients to cover for personnel who were sick.

#### Worry about infecting patients, co-workers, and oneself

The RN and MICM-physicians at work during the pandemic were scared of infecting patients, co-workers, and themselves. One of the RNs described how a MICM-physician had decided to resign as an MICM-physician because of their discomfort making home visits during the pandemic. Some MICM-physicians were in the stated risk groups of being severely ill if infected with COVID-19 and therefore avoided seeing patients due to worry about being infected. The participants explained that at the beginning of the pandemic they were not allowed to wear protective gear in the form of masks or face shields when in close contact with patients because of the scarcity of protective gear, which worried them. The authorities had stated that this protective gear was unnecessary when working with older patients, and there was a nationwide lack of protective gear. The participants explained how they had been told that protective gear needed to go to hospitals instead, because of the scarcity of it. This lack of protective gear prevented the RN and MICM-physicians from making home visits, since they feared that they might infect the older patients. The COVID-19 virus was described as devious, and the RN and MICM-physicians did not want to endanger the patients, their co-workers, or themselves by not wearing protective gear. Some participants bought their own protective gear when it was not supplied by the municipality or primary health care center. When the protective gear was allowed to be worn, the participants were relieved since it was a relief easing the worry of infection. A MICM-physician said: *“We always work with face shields now. We’ve followed the authorities’ regulations. We didn’t use face shields in the beginning, but if we had, maybe we could have avoided infection.”,* relating this to the patients who had been infected with COVID-19 from the care personnel. After protective gear was permitted, the way RNs and MICM-physicians worked changed. Protective gear was used constantly when the RNs and MICM-physicians were within two meters of a person during work, and the participants became less worried about infecting someone or being infected themselves. The RNs and MICM-physicians said that the patients found the protective gear odd at first but that everyone soon got used to how the participants looked. However, the protective gear was described as impacting communication with the patients, as the patients struggled to hear what the RNs and MICM-physicians said.

To prevent transmission, the participants explained that they followed the authorities’ restrictions, even if the recommendations changed often. The changing directives were seen as challenging, but the RNs and MICM-physicians worried less about transmission if they knew that they were following up-to-date recommendations. The MICM-physicians or RNs held information or visited personnel meetings to answer questions from the ANs about COVID-19, as the participants noticed that the ANs needed additional knowledge to ease their worries about infecting others. The information was described by the participants as valuable for all working in home health care. The RNs wanted the ANs to feel that the RNs knew what they were doing, even if they were not always sure themselves because of the changing directives. One RN said: *“We want the personnel to feel safe when they work with the patients, to not be afraid. We got this.”*

According to the RNs and MICM-physicians, some patients did not worry about the virus itself. Other participants described how some patients declined help from home care, since they worried that the ANs would infect them. The RNs and MICM-physicians explained how they had tested many patients for COVID-19. The patients were tested when they changed care units, such as coming home from hospital or if the patients had spent time at a short or long-term care facility, as well as when they had symptoms. The participants explained that they had been liberal with testing to ensure that the virus did not spread and to ease worry. The RNs and MICM-physicians took great pride in having a patient group that had not been infected by COVID-19 and spoke about how they had tried their hardest to constrain infectivity by following the changing restrictions.

#### Worry about maintaining provision of care due to colleagues’ increased sick leave

The participants worried about the increasing number of personnel on sick leave. The ANs were especially affected and had to visit more patients than usual each shift. This resulted in decreased continuity for the patients, according to the RNs. One RN said: *“Personnel have been on sick leave, which has impacted continuity for the patients, since planning has to be changed, and there’s a lack of personnel.”* The RNs were also affected by the restrictions and added sick leave, with fewer RNs to treat the patients. The increased sick leave meant that daily plans had to be changed every morning to cover for the RNs who were home. The increased sick leave also meant that vacation days could not be used because home health care was low on personnel. Not being able to use vacation days created discontent, since the ANs and RNs were tired from covering more shifts, according to the RNs.

### Trying to bridge the gap of patients’ isolation

Patients became isolated during the pandemic, having to stay at home to avoid becoming infected, according to the RNs and MICM-physicians. Social meeting places closed, next of kin did not dare visit, and the patients avoided doing errands. The isolated patients’ health was recognized as declining when they became lonely and inactive. The participants tried to bridge the gap of isolation to support the patients but felt powerless against the loneliness the patients expressed. The next of kin were described as feeling worried and guilty for not visiting the isolated patients. The RNs and MICM-physicians tried to alleviate this increased worry by bridging the gap through added information and phone communication.

#### Powerless in the face of declined health for isolated patients

The RNs and MICM-physicians described how the patients’ health had declined during the pandemic, resulting in a sense of powerlessness for the RNs and MICM-physicians. The participants explained that the patients’ health and wellbeing were impacted by isolation rather than fear of COVID-19. The next of kin did not dare visit the patients due to the pandemic, because they were scared of infecting the. The patients were described as sad, and they longed to see their next of kin. One RN said: *“They’ve become even more isolated. It was boring before, and now it’s even more boring.”* Holidays especially affected the patients. The loneliness expressed by the patients was described as a challenge for the participants where they struggled to leave patients when visiting. Patients refused to eat and found their isolated life boring, something the participants felt powerless to stop. The pandemic was described as impacting the patients’ physical and mental health, since the patients mostly stayed inside and became inactive. The RNs tried to encourage patients to go outside and go for a walk but discouraged them from approaching crowded areas.

The participants explained that they often became the only persons the patients met. COVID-19 was often the predominant topic the patients spoke about because of its impact on their lives. The RNs and MICM-physicians described trying to support the patient and talk about other things, but limited time restricted how they could impact patients’ daily lives, adding to their sense of powerlessness. Working in protective gear was described as less personal by the participants, and the patients sometimes struggled to hear the RNs and MICM-physicians when they were wearing the protective gear. One MICM-physician said*: “We’ve lost some contact. It’s hard to create that alliance with the patient with all the protective gear.”* The patients who had less help from home health care were those described by the participants as having been most affected, as they had an active social life before the pandemic. Those who had more help were isolated before the pandemic because of their worse health.

The RNs and MICM-physicians noted that older persons’ social meeting places had closed because of the pandemic, which negatively influenced their health and wellbeing. Other social interactions that paused were activities such as an AN visiting the patient’s home for a walk or sitting with them to eat. Staying away from other social interactions, such as going shopping, was also viewed as something the patients perceived as isolating, which worsened their health and wellbeing, the participants explained. The RNs commented that the ANs tried to alleviate the patients’ isolation but were limited in how much they could help because they could not give the patients more time than was individually approved by the municipal social care.

#### Meeting increased worry and guilt from the next of kin

The next of kin were described by the participants as worrying about their relatives during the pandemic. Because of this, they called mainly the RNs more often to ease their worry. Increased phone communication became a way for the RNs to bridge the gap between the patient and their next of kin and to ease next of kin guilt. The experience of increased phone contact from worried next of kin varied among the municipalities, where the RNs, MICM-physicians and ANs group received increased phone calls in different municipalities. The participants said that even if the restrictions stated that the next of kin should not visit their relatives, some next of kin still did because of their sense of guilt. Regardless of their personal opinion, the RNs could not control who visited patients’ homes. One RN said: *“Some have visited their loved ones regardless of whether they’re old and sick, and I just hope they’ve kept a distance, but I can’t be in the home and see how they act.”* The RNs and MICM-physicians commented that some next of kin followed the restrictions and had not seen their relatives for several months, which led to increased worry and guilt for the next of kin. The participants became a bridge between the next of kin and the patient, as the RNs and MICM-physicians became the eyes of the next of kin who could not visit. The next of kin were described as feeling guilty for not seeing the patient. One RN said: *“There are a lot of next of kin conversations, and they’re worried about their parents; they feel guilty for not visiting.”* Because of this sense of guilt, the next of kin required more in-depth information about the patient, according to the RNs. This increased need for information led to the RNs spending more time on the phone than before the pandemic, trying to bridge the forced gap between the patient and next of kin.

## Discussion

Working in the COVID-19 pandemic as a RN or MICM-physician in home health care was described as having resulted in a forced change to the ways of working, which has also been expressed by health care professionals working in hospitals [31]. Health care professionals working in a MICM during the pandemic described how they were asked only to make necessary visits to the patients, which they struggled to differentiate. Instead of making several visits, the RNs tried to fit as much as they could into one visit. Health care professionals working in hospitals and primary health care have also described this type of cluster care, where they tried to reduce the time they spent with the patient [31, 32]. The RNs described how the MICM-physician became invisible in the care, since the MICM-physician no longer made home visits. Similar findings have been described where the RNs noted significant changes in the delivery of care, including taking on the roles of several health care professionals, since other professions did not want to visit infected patients [31]. This could explain why RNs perceived a significantly higher workload compared to other health care professionals during the COVD-19 pandemic [21]. Physicians working in primary health care settings have reduced their in-person consultations with patients [33], similar to the MICM-physicians pausing their home visits.

The forced pause in the MICM’s usual work was described as having made home health care need-oriented, rather than person-centered, which the MICM is grounded in [17, 18]. Person-centered care requires a partnership between the health care personnel and the patient [19, 20]. Person-centered communication has been described as crucial in responding to the pandemic when caring for older persons [34]. The lack of home visits, the MICM-physician pausing annual visits, and the pause in creating and updating medical health care plans could be seen as ways that person-centered care became lacking during the pandemic in the MICM. Due to the COVID-19 pandemic, health care has been forced to switch focus back to a disease, stepping away from a patient focus [35]. RNs have been described as striving for person-centered care despite the pandemic’s obstacles [36]. Likewise, in this study, the RNs and MICM-physicians struggled when they could not create a partnership as they usually did because of the paused home visits.

The participants explained how they had been forced to use digital and phone communication because of the pandemic despite preferring in-person meetings. Digital and phone communication was described as negatively influencing the quality of care, since misunderstandings between the RNs and MICM-physicians resulted in more work. Increased use of digital tools for communication has been seen during the pandemic, when digital inequalities may have been further reinforced by the lack of access to digital support [37], which is often limited in the home health care setting. Communication with patients digitally or by telephone was also described by the participants as a challenge, in contrast to a study of general physicians, who were satisfied with telephone consultations [38]. Digital tools can, however, be a challenge for older persons to use, even when they are the sole tool for social interaction, as during the pandemic [6]. The RNs and MICM-physicians expressed a sense of capitulation in being forced to use digital and phone communication. They commented on how they had no choice but to work this way, regardless of whether they felt that it negatively impacted the quality of care. As the world heavily relies on digital technology for communication, less experienced people may need more support than ever before because of the changed ways of working due to the pandemic [37]. Research on digital support in home health care and the MICM, as well as the experiences of in-person versus digital communication in health care, is lacking and could be further explored. It is crucial to learn from the digital adversity seen in health care during the pandemic and to substantially embed this knowledge into the care models of the future [39].

The pandemic brought worry about illness into the work setting. The participants described being worried about infecting patients, co-workers, and themselves, which supports previous studies from other health care settings [27, 31, 36, 40, 41]. One MICM-physician ended their employment because of this worry. Other MICM-physicians, who were in the risk group of being severely ill if infected, avoided seeing patients. This concurs with previous studies, where health care professionals with underlying health concerns were particularly worried about their safety while working in health care during the pandemic [31, 32]. Lack of protective gear was described by the RNs and MICM-physicians at the beginning of the pandemic, when hospital care was prioritized over home health care. The lack of protective gear made the participants feel at risk of being infected, which has also been noted by RNs and MICM-physicians working outside hospital settings during the pandemic [27, 32, 33, 42]. Health care professionals working in hospital settings also described a lack of protective gear and an unwillingness to share protective gear for fear of future shortages [31]. Caring in protective gear has been described as uncomfortable. The face shields cause headaches, and respiratory protection could lead to shortness of breath [36]. Discomfort from the protective gear was not something the RNs and MICM-physicians described in the present study, it was rather a relief when they were allowed to use them. Before the employer supplied protective gear, RNs and MICM-physicians bought their own, as described by other home care personnel [27]. The constantly changing restrictions became a way for the health care professionals working in the MICM to feel like they were preventing contagion, even if the continuous changes were challenging. This finding has been supported by several studies in which health care professionals described how changing recommendations, sometimes within hours, caused uncertainty and anxiety [27, 31, 36, 40]. Lack of knowledge about the virus made the RNs feel insecure, but they still tried to convey knowledge to the ANs and support them as best they could. Working during the COVID-19 pandemic has been described as a “learn as you go” experience with no additional training [27, 31]. Lack of knowledge was a source of interest for some RNs, even if they experienced insecurities in their limited knowledge [36], something the RNs in the present study also experienced. A sense of pride in having cared for the patients and keeping COVID-19 at a distance was described by the RNs and MICM-physicians, which is in line with other studies [31, 36]. This sense of pride could extend beyond patient care, with health care professionals expressing their realization of being capable of more than before [36, 40]. This was, however, not described by the RNs and MICM-physicians in the present study; their pride focused on their patients not becoming infected.

The participants worried about upholding health care provision because of the increasing number of personnel on sick leave, which has been found previously [31, 41]. The pandemic has led to RNs experiencing heavier workloads [21] and distress related to quality of care and patient safety to the point of considering leaving their employment [23]. The RNs in the present study described increased workloads because of colleagues being on sick leave, as well as being unable to take vacation days, which led to discontent. This discontent may in the long run also lead to RNs leaving their employment, which would result in further personnel shortages in home health care, which already suffers from a nationwide shortage of educated personnel [12, 13].

The participants described how they felt powerless in the face of their patients’ declining health and explained that they tried to bridge the gap caused by isolation during the pandemic. The patients admitted to home health care lost several social contexts, including attending meeting places, seeing their next of kin, or running errands. The pandemic’s impact has been described as more severe on adults in municipality care than the rest of the population [41]. Losing a sense of connection can change a person’s perception of the world, and social isolation and loneliness have a greater risk of mortality than smoking or obesity [43, 44]. Isolation and declining health threaten the patient’s life [6]. The RNs described how they and the ANs became the only persons the patients met, which made it a struggle to leave the patients when they felt lonely. RNs working in a hospital setting expressed similar feelings, experiencing becoming the sole emotional support for fearful patients [31]. Working in protective gear was further described as adding a sense of lost contact with the isolated patients, which was described similarly by RNs working in other fields [31, 34, 36]. Losing physical touch due to the barriers of protective gear and distancing can harm the positive, trusting relationship needed in the delivery of high-quality care [45]. Bridging the gap between the isolated patients and their next of kin meant that the participants had to deal with the next of kin’s increased worry and guilt. The RNs and MICM-physicians described how the next of kin mostly did not visit the patients, which led to increased worry and guilt, something also seen in next of kin in hospital settings [31, 36]. The participants described becoming the next of kin’s eyes, since they could not visit their relatives out of fear of infecting them. The participants described how contact with the next of kin happened mostly through telephone consultations, which was observed by other health care professionals keeping in contact during the pandemic [36, 46]. The RNs and MICM-physicians therefore did their best to bridge the gap of isolation for the patients, despite the forced change in their way of working as well as the worry about illness in the work setting.

### Methodological considerations

The purpose of this study was to explore the experiences of RNs and physicians in an MICM working in home health care during the COVID-19 pandemic. RNs and MICM-physicians working in a MICM in home health care were seen as a relevant group to the study aim and were therefore chosen for qualitative data collection through interviews. The participants came from different municipalities and primary health care centers, giving a diverse view of experiences working in home health care during the pandemic. One limitation was that all the participants worked in a MICM, so their experiences may differ from those of others working in home health care. This can, however, enable comparisons to explore if experiences differ between health care professionals working in home health care but not in MICM.

The main focus of the interviews was not COVID-19 but was part of a larger study on health care professionals experiences of working in an MICM [47, 48]. The participants were informed prior to the study that questions about COVID-19 would be included. This could mean that the participants did not have any particular bias in talking about the COVID-19 pandemic. The study findings were evaluated in terms of trustworthiness and demonstrated credibility, dependability, and transferability [49]. The study’s credibility is strengthened by the choice of participants, the data collection method, the presentation of quotes in the findings, and the description of the analysis process. The dependability was enhanced through discussions in the research group and throughout the analysis. Transferability has been enabled through careful description of the study population, data collection, data analysis, and setting.

## Conclusion

The RNs and MICM-physicians working in home health care during the COVID-19 pandemic faced a change in their way of working, which showed the intricacy of building relationships with the older patients which they cared for. The RNs and MICM-physicians described how relationship building and maintaining became difficult when frequent visits and physical touch was impossible. The forced pause also resulted in the disruption of the work of improving health care. Furthermore, the pandemic resulted in communication being moved from physical meetings to be conducted over the phone or video call, which led to more work due to miscommunication and therefore influenced the quality of care. The worry of infecting other as well as becoming infected themselves led to the RNs and MICM-physicians spending their own money to buy protective gear, since it was not provided by the employers, which further shows how the hospital personnel and patients were prioritized over the home health care personnel and patients. Additional sick leave increased worry of upholding the provision of health care, and being unable to use vacation days resulted in a feeling of discontent among the RNs. The patients were said to have become isolated, described by the participants to influence patients’ physical and mental health, something the RNs and MICM-physicians felt powerless to stop. Furthermore, they felt powerless in the face of the increasing worry and guilt among the next of kin. These lessons should be considered when planning future care and conduction research within the area. Specifically, the relationship building aspect of quality care for older persons, and the possibilities and challenges with phone and video communication between health care personnel in different organizations should be further explored.

## Data Availability

The data sets analyzed during the current study are not publicly available due to ethical principles and the guidelines of the Swedish Ethical Review Authority, but data not comprising confidential information are available from the corresponding author on reasonable request.

## Abbreviations

MICM: Mobile Integrated Care Model
RN: Registered nurse
AN: Assistant nurse
MICM-physician: Home health care physician
